# Supplementary material for: RAS oncogenic activity predicts response to chemotherapy and outcome in lung adenocarcinoma
Source: Nat Commun. 2022 Sep 26;13:5632. doi: 10.1038/s41467-022-33290-0 (PMC9512813; doi:10.1038/s41467-022-33290-0)
Supplement: Supplementary file 1 — Supplementary Information [file 41467_2022_33290_MOESM1_ESM.pdf]

Supplementary figure 1

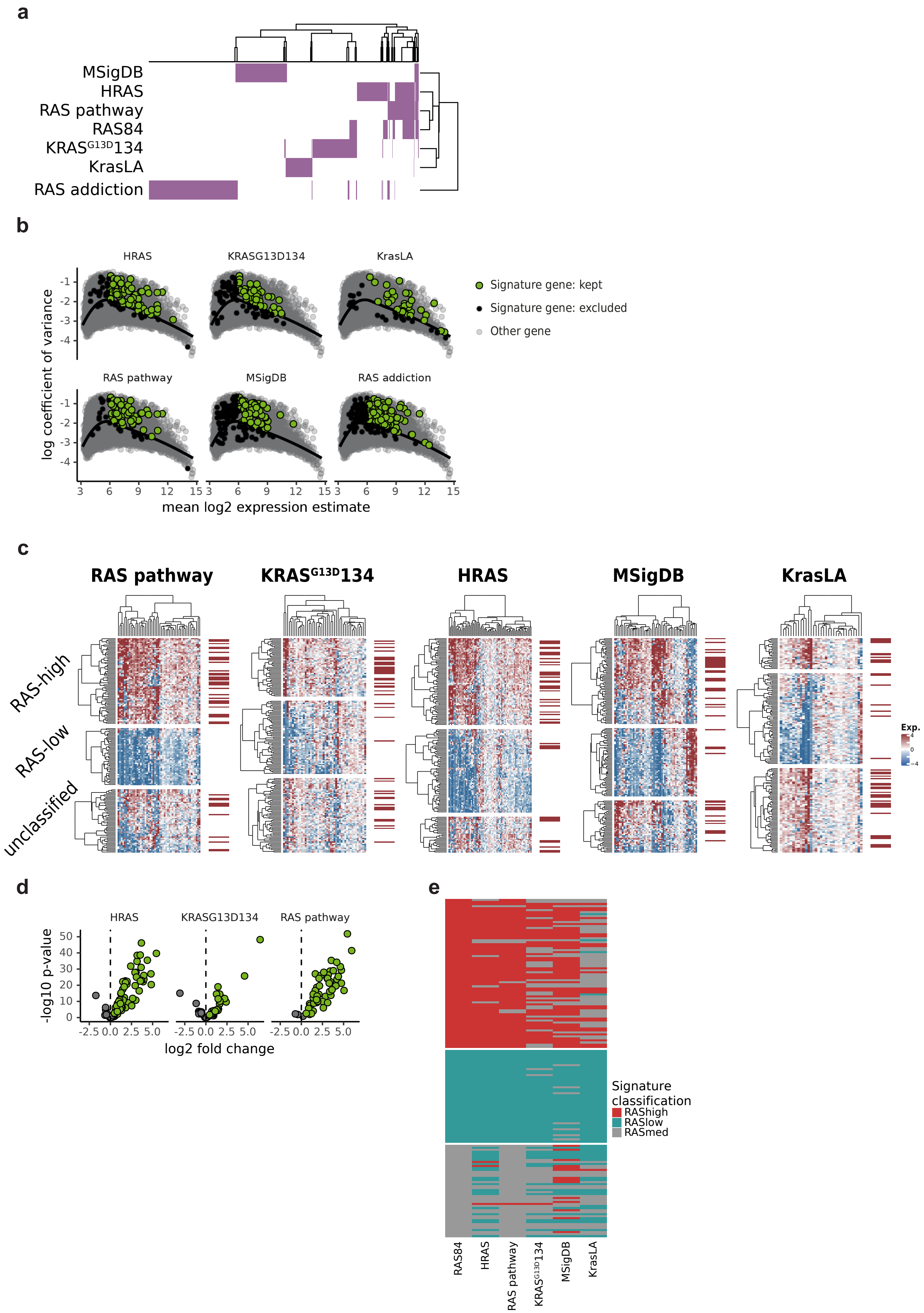

Supplementary figure 1

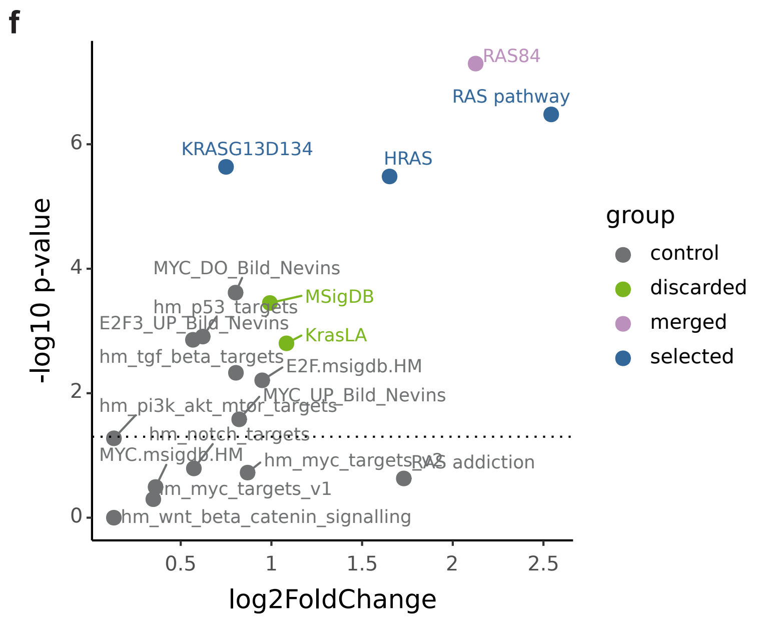

Supplementary Figure 1. (a) A heatmap showing the degree of overlap between the different RAS gene signatures. The genes are represented by the columns. Blue indicates the presence of a gene in a given signature. **(b)** Scatter plots highlighting RAS signature gene selection in the context of CCLE lung cell line expression data. Selected signature genes are highlighted in green, discarded in black and non-signature genes in grey. Log coefficient of variance was plotted against mean VST expression and a loess curve fitted to the data, shown by the black line. Signature genes with positive residuals with respect to the loess fit and a VST mean value > 6 were selected. **(c)** Heatmaps showing filtered RAS gene signatures (columns) across filtered (see methods) CCLE lung cell line expression data (rows). The expression of each gene across the cell lines is scaled to the median value. The cells and genes are clustered using hierarchical clustering with ward.D2 agglomeration. The cell lines are grouped by segmentation of the cluster dendrogram into three groups. The groups are labelled as RAS-high, RAS-low and unclassified based on the mean signature expression across the group. KRAS mutational status is indicated by the red bars to the right of the heatmaps. **(d)** Volcano plots showing the difference in expression between RAS-high and RAS-low groups for HRAS, KRASG13D134 and RAS pathway signatures. Genes with an fdr < 0.05 and a positive log2 fold change in the RAS-high group were selected, shown in green (Limma analysis). **(e)** A Heatmap showing CCLE cell line classification by the different signatures. The cell lines are grouped by RAS84 labelling. **(f)** A volcano plot highlighting signature performance. The y-axis shows the significance of KRAS mutation segregation across RAS-high and RAS-low activity groups (-log10 p-value, chi-square). The x-axis shows the log2 fold change in mean expression between RAS-high and RAS-low activity groups. RAS84 meta signature is shown in purple, selected parent signatures in blue and discarded signatures are in green. A selection of RAS related control gene signatures are shown in grey, highlighting the specificity of the selected RAS signatures to measure RAS activity.

Supplementary figure 2

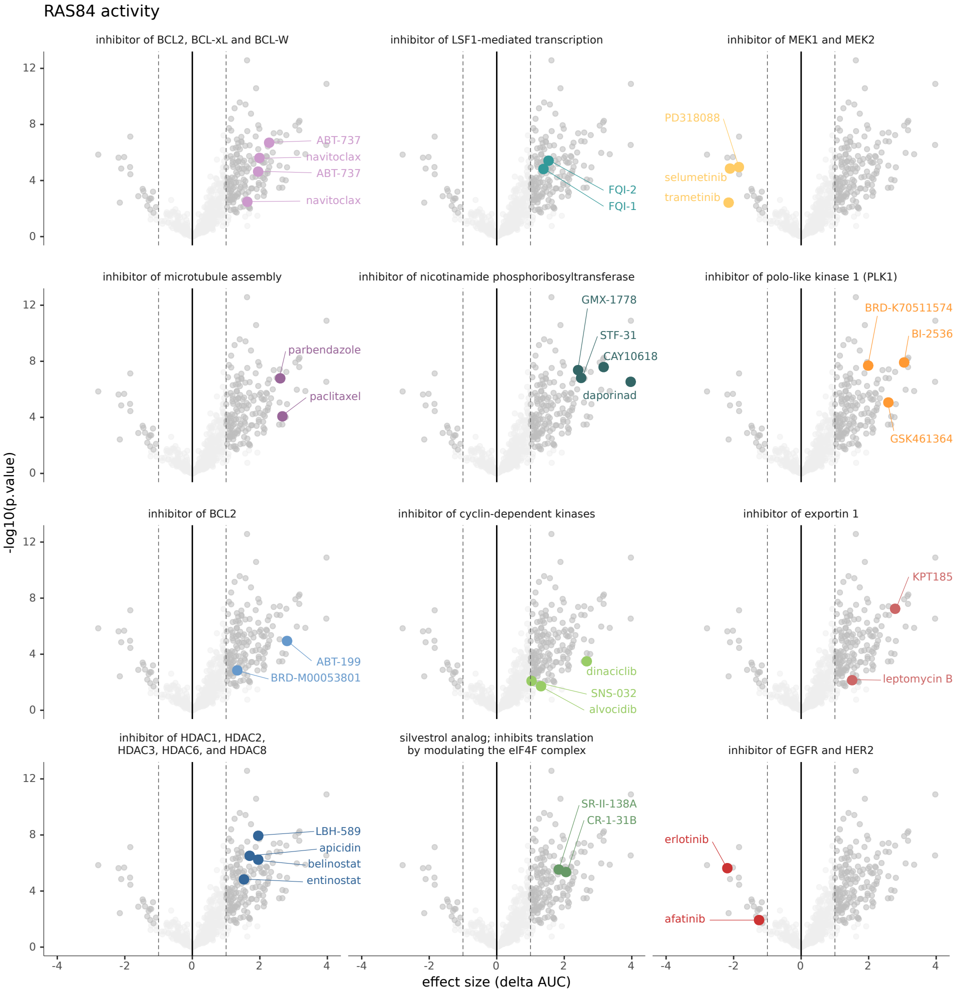

Supplementary Figure 2. Volcano plots showing differences in IC50 values between RAS high and low CCLE cell lines. Drugs with enriched target annotations in the significant sensitive and resistant groups are highlighted. Drugs with an absolute log2 fold change > 1 and fdr < 0.05 are shown in dark grey. Results from both GDSC1 & 2 are shown (Linear model, Benjamini-Hochberg FDR correction)

Supplementary figure 3

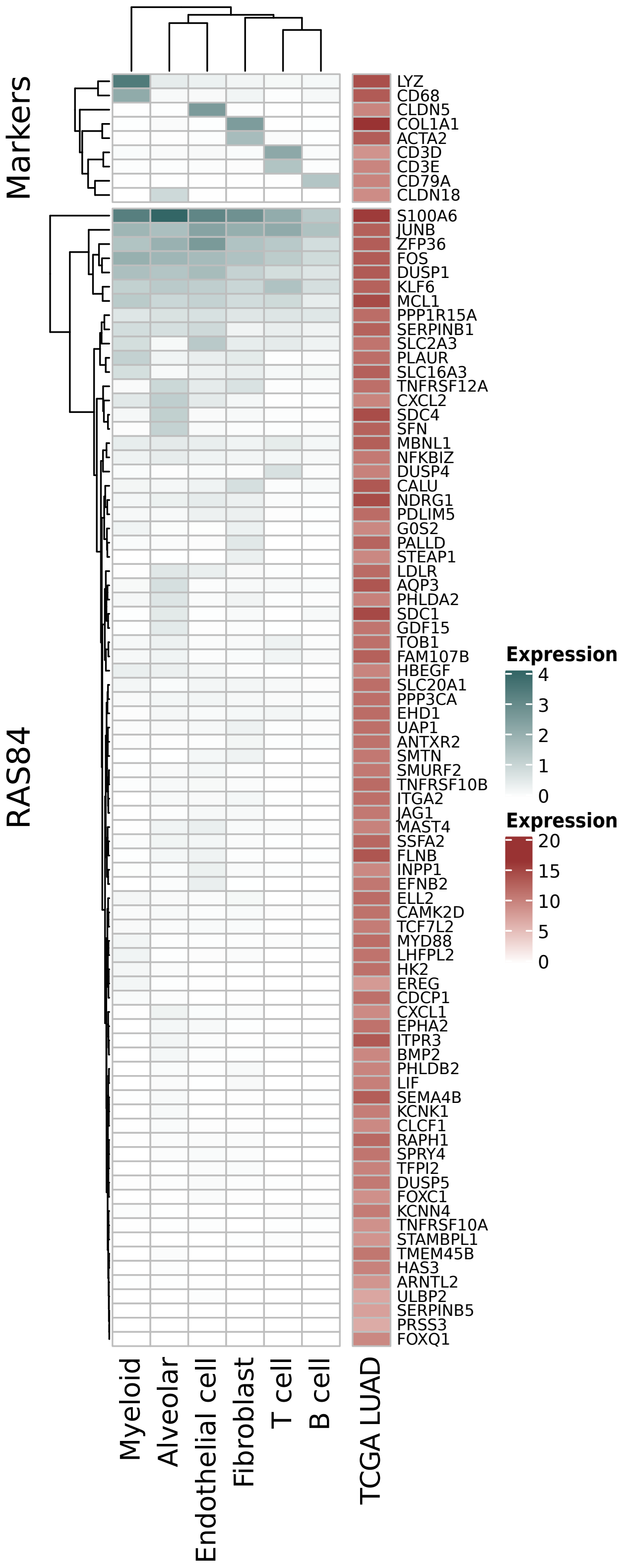

Supplementary Figure 3. A heatmap showing minimal RAS84 expression detected in tumour infiltrating cells. The heatmap shows mean log2 CPM RAS84 expression across tumour infiltrating cell types from five NSCLC samples. The heatmap to the right shows mean VST normalised RAS84 expression across TCGA LUAD cohort. For reference a set of infiltrating cell expression markers are shown at the top.

Supplementary figure 4

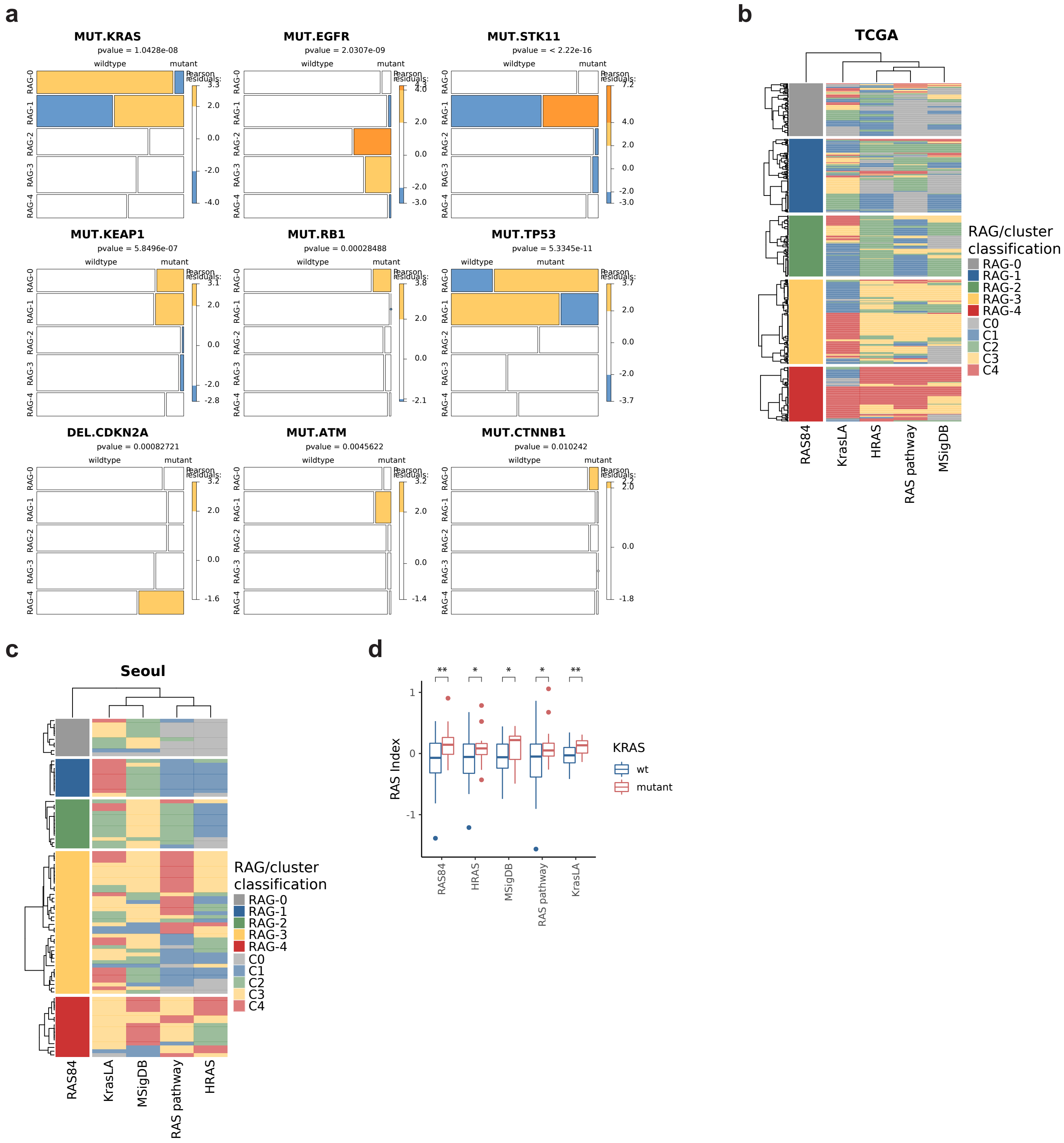

Supplementary Figure 4. (a) Mosaic plots showing contingency table frequencies for given genetic variants across TCGA LUAD RAGs. The chisq test p-value is shown at the top of each plot. The plots were generated using the mosaic function from the vcd R package. The widths and heights of the rectangles represent the relative frequencies in each group. The colours are derived from the Pearson residuals and highlight over (orange) and under (blue) represented groups. **(b)** Heatmap showing the overlap between RAG labels assigned by clustering to the TCGA LUAD patients using RAS84 and the cluster defined using the published signatures. Samples labelled by clustering using published signatures are represented in lighter colours. **(c)** Heatmap showing the overlap between RAG labels assigned by clustering to the Seoul cohort patients using RAS84 and the cluster defined using the published signatures. Samples labelled by clustering using published signatures are represented in lighter colours. **(d)** RI and mean public signature expression value distributions across the Seoul cohort, segmented by KRAS mutation status (KRASmut n=18, WT n=69; ** P≤0.01, * P≤0.05, n.s. = P>0.05, linear model). The boxes show the median and IQR, the whiskers indicate +/- 1.5 x IQR, outliers lie outside this range.

Supplementary figure 5

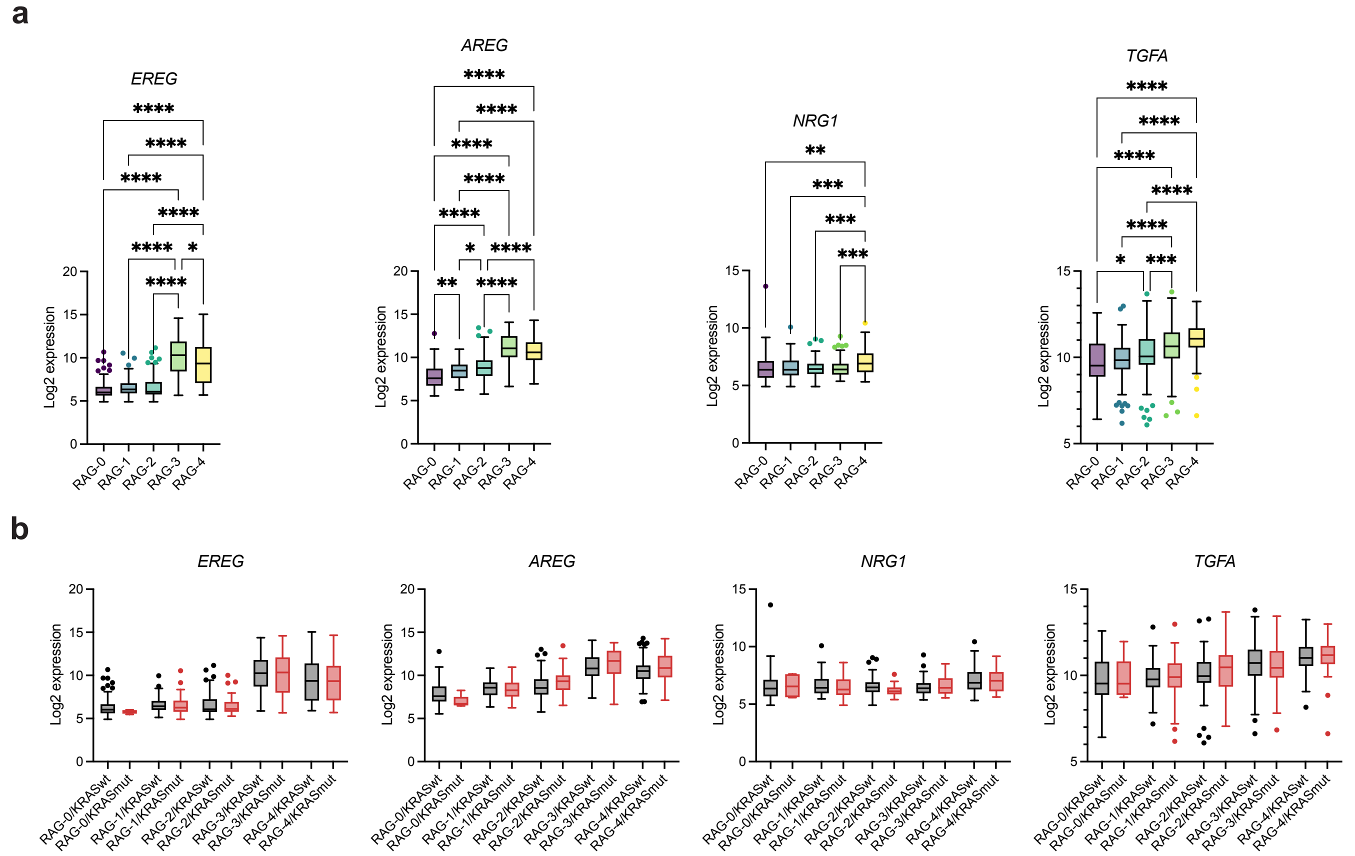

Supplementary Figure 5. (a) Tukey box and whisker plot with median (centre line) and outliers (dots) showing log2 expression levels of indicated genes across RAGs in LUAD TCGA. RAG-0/KRASwt (n=76), RAG-0/KRASmut (n=5), RAG-1/KRASwt (n=59), RAG-1/KRASmut (n=54), RAG-2/KRASwt (n=72), RAG-2/KRASmut (n=22), RAG-3/KRASwt (n=89), RAG-3/KRASmut (n=41), RAG-4/KRASwt (n=52), RAG-4/KRASmut (n=32). ANOVA (FDR<0.01), **** P≤0.0001, *** P≤0.001, ** P≤0.01, n.s. = P>0.05. **(b)** Tukey box and whisker plot with median (centre line) and outliers (dots) showing log2 expression levels of indicated genes across RAGs separated into KRASwt (grey) and KRASmut (red) in LUAD TCGA. RAG-0 (n=81), RAG-1 (n=113), RAG-2 (n=94), RAG-3 (n=130), RAG-4 (n=84). ANOVA (FDR<0.01), **** P≤0.0001, *** P≤0.001, ** P≤0.01, n.s. = P>0.05.

Supplementary figure 6

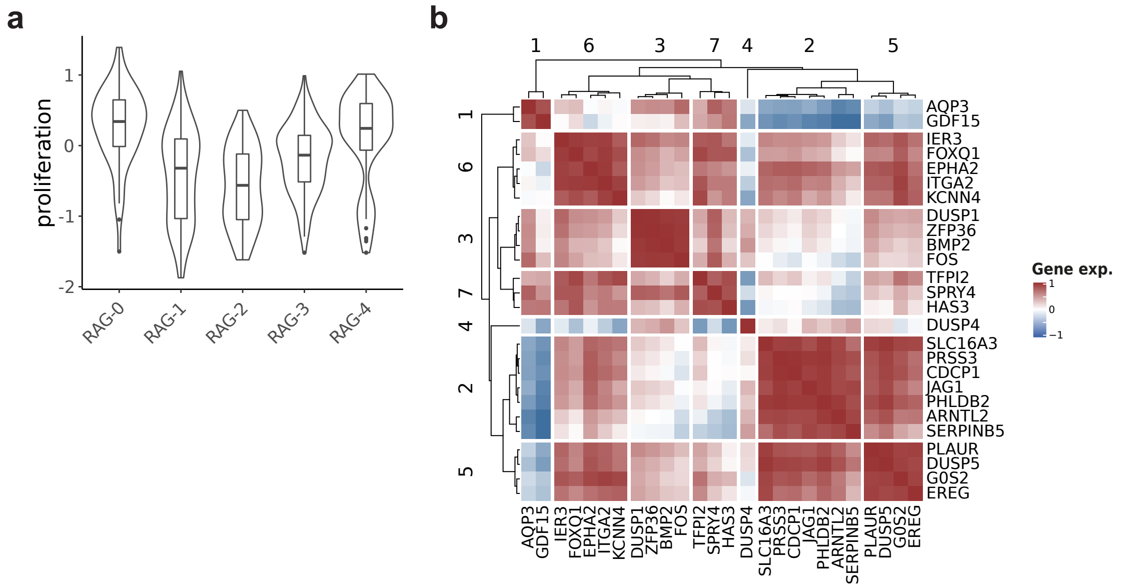

Supplementary Figure 6. (a) Distributions of TCGA LUAD sample proliferation scores per RAG. The proliferation score was taken from the TCGA pancancer data release (RAG-0 n=81, RAG-1 n=113, RAG-2 n=94, RAG-3 n=130, RAG-4 n=84). The boxes show the median and IQR, the whiskers indicate +/- 1.5 x IQR, outliers lie outside this range. **(b)** Heatmap showing Pearson's correlation coefficients between RAS84 gene expression VST z-scores across the TCGA LUAD cohort. RAS84 genes with a deviation of > 1 log2 from the median VST value were selected. The genes were hierarchically clustered using ward.D2 agglomeration. The genes were grouped by segmentation of the dendrogram into seven correlated groups.

Supplementary figure 7

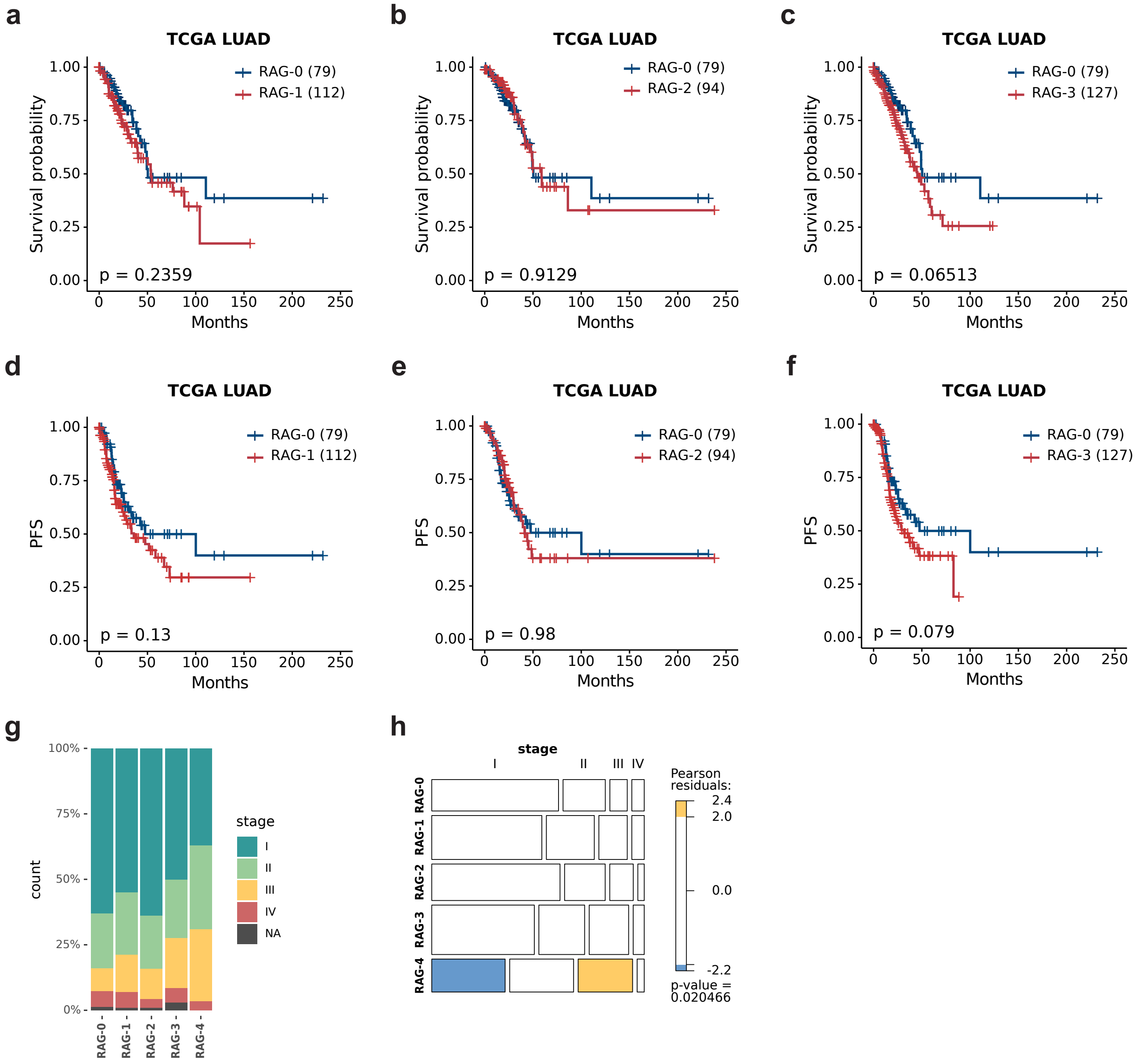

Supplementary Figure 7. (a) Kaplan-Meier plot showing overall survival data from the TCGA LUAD cohort for patients from RAG-1 and RAG-0 (coxph p-value 0.2359), the number of patients per group is indicated in brackets. **(b)** Kaplan-Meier plot showing overall survival data from the TCGA LUAD cohort for patients from RAG-2 and RAG-0 (coxph p-value 0.9129), the number of patients per group is indicated in brackets. **(c)** Kaplan-Meier plot showing overall survival data from the TCGA LUAD cohort for patients from RAG-3 and RAG-0 (coxph p-value 0.6513), the number of patients per group is indicated in brackets. **(d)** Kaplan-Meier plot showing progression-free data from the TCGA LUAD cohort for patients from RAG-1 and RAG-0 (coxph p-value 0.2359), the number of patients per group is indicated in brackets. **(e)** Kaplan-Meier plot showing progression-free data from the TCGA LUAD cohort for patients from RAG-2 and RAG-0 (coxph p-value 0.2359), the number of patients per group is indicated in brackets. **(f)** Kaplan-Meier plot showing progression-free data from the TCGA LUAD cohort for patients from RAG-3 and RAG-0 (coxph p-value 0.2359), the number of patients per group is indicated in brackets. **(g)** Stage percentages per RAG in TCGA LUAD. **(h)** Mosaic plot showing contingency table frequencies for TNM stage across TCGA LUAD RAGs with chisq test p-value. The widths and heights of the rectangles represent the relative frequencies in each group. The colours are derived from the Pearson residuals and highlight over (orange) and under (blue) represented groups.

Supplementary figure 8

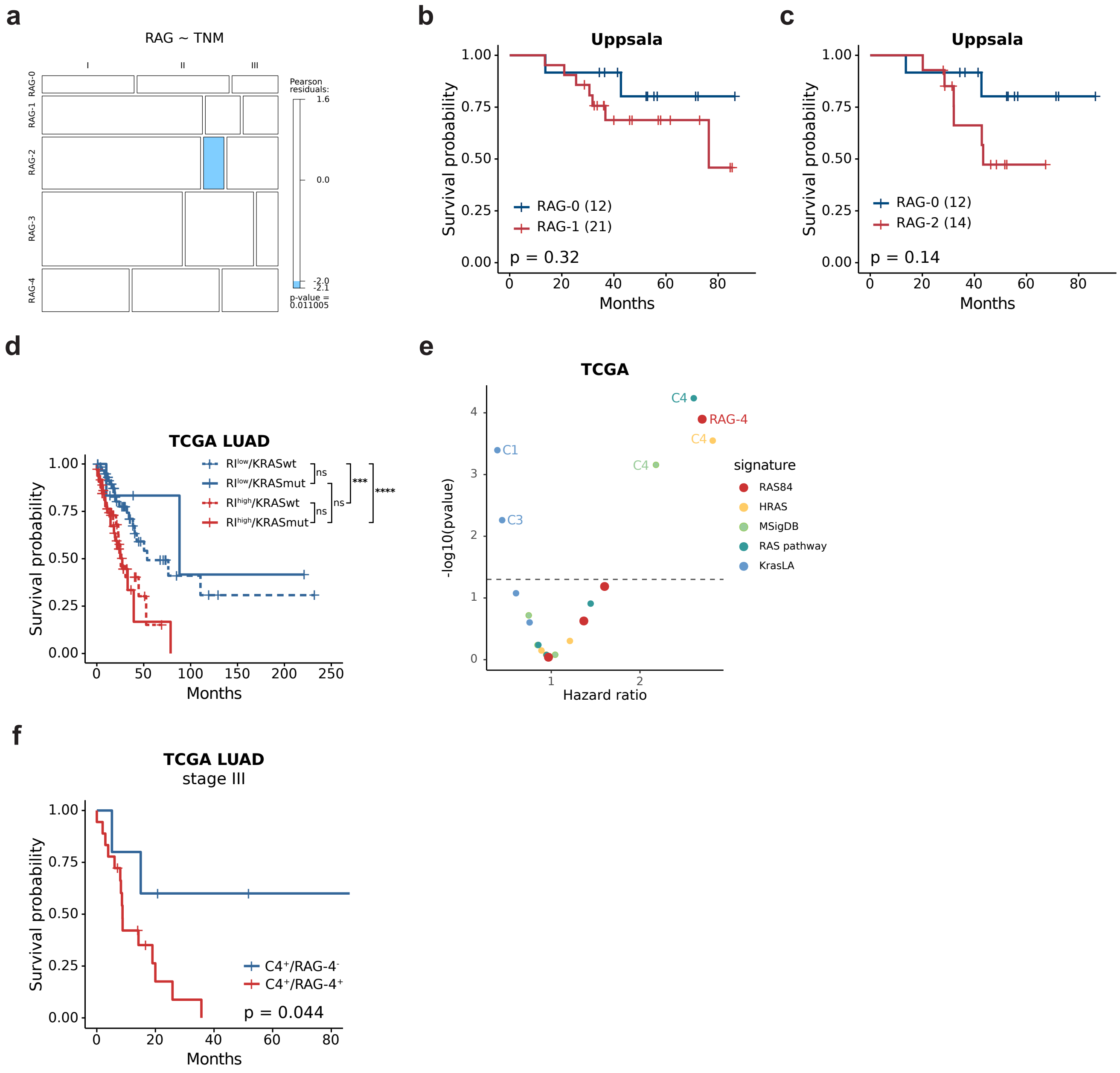

Supplementary Figure 8. (a) Mosaic plot showing contingency table frequencies for TNM stage across Uppsala RAGs with chisq test p-value. The widths and heights of the rectangles represent the relative frequencies in each group. The colours are derived from the Pearson residuals and highlight over (orange) and under (blue) represented groups. **(b)** Kaplan-Meier plot showing overall survival data from the Uppsala cohort for patients from RAG-1 and RAG-0 (multivariate coxph p-value 0.32), the number of patients per group is indicated in brackets. **(c)** Kaplan-Meier plot showing overall survival data from the Uppsala cohort for patients from RAG-2 and RAG-0 (multivariate coxph p-value 0.14), the number of patients per group is indicated in brackets. **(d)** Kaplan-Meier plot showing TCGA LUAD RI high and low patients stratified by KRAS mutation status. RI high represents the upper tertile, RI low the lower tertile across all RI patient values. **(e)** Multivariate Cox Proportional Hazards test p-values and hazard ratios plotted for RAGs derived from RAS84 and clusters derived from the published signatures. The p-values are plotted on a -log10 scale. **(f)** Kaplan-Meier plot showing overall survival in stage III TCGA LUAD patients stratified by concordance between RAG-4 (RAG-4) and RAS-pathway signature (C-4) (p-value 0.044).

Supplementary figure 9

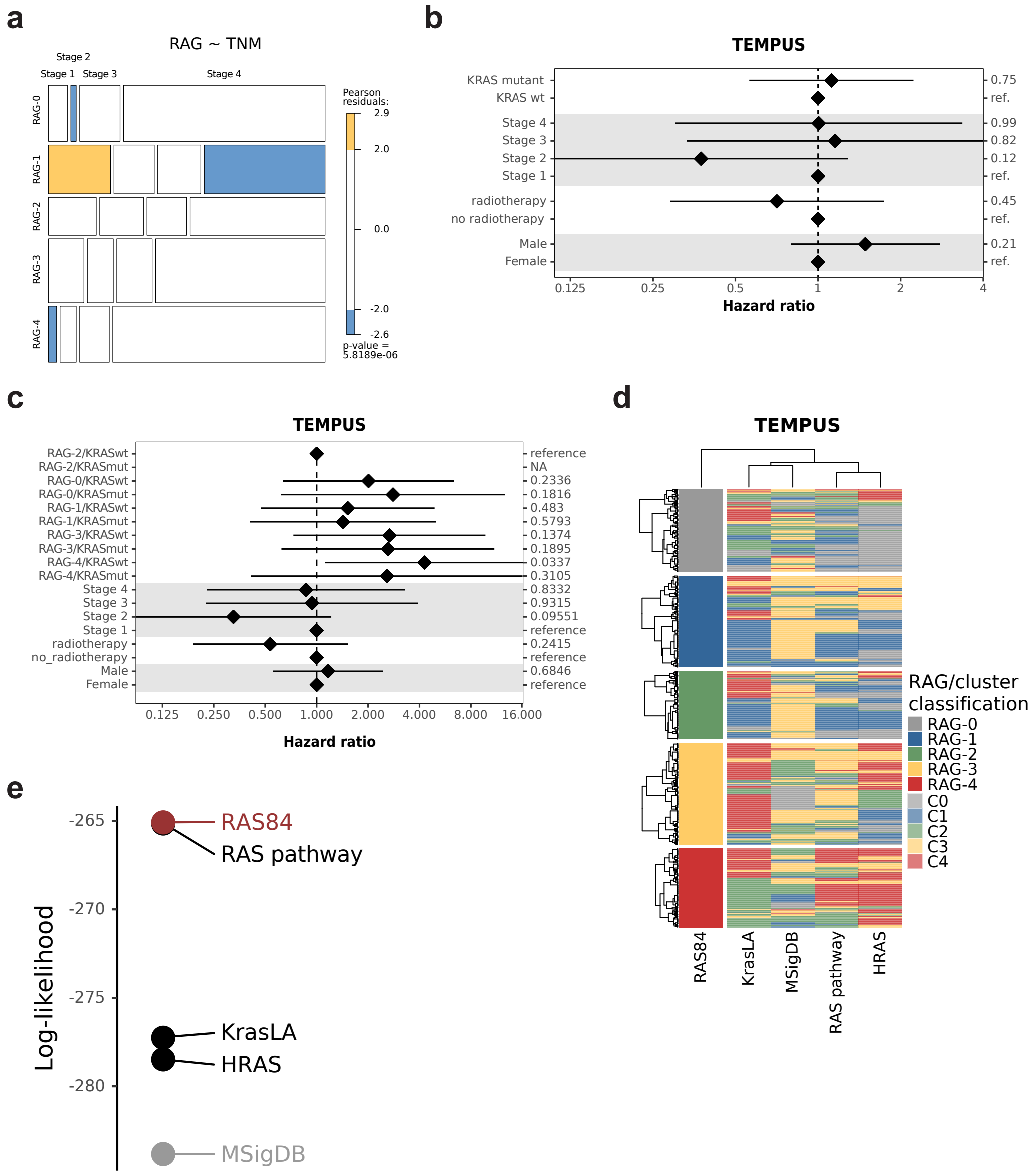

Supplementary Figure 9. **(a)** Mosaic plot showing contingency table frequencies for TNM stage across TEMPUS RAGs with chisq test p-value. The widths and heights of the rectangles represent the relative frequencies in each group. The colours are derived from the Pearson residuals and highlight over (orange) and under (blue) represented groups. **(b-c)** Forest plots showing results from a multivariate Cox proportional-hazards analysis of PFS after chemotherapy in the TEMPUS lung adenocarcinoma cohort (n = 100 patients). KRAS mutants are compared to KRAS wt (b). Groups defined by RAG classification and KRAS mutational status are compared to RAG-2/KRASwt Due to the small number of patients in this cohort, group RAG-2/KRASmut did not have patients (c). **(d)** Heatmap showing overlap between RAG labels assigned by RAS84 SVM classification and clustering using the published signatures in the TEMPUS cohort. **(e)** Log-likelihood values from a GLM fit (family=binomial) of KRAS mutation status across the 5 RAG groups derived using RAS84 (SVM classification) and the published signatures (clustering).

Supplementary figure 10

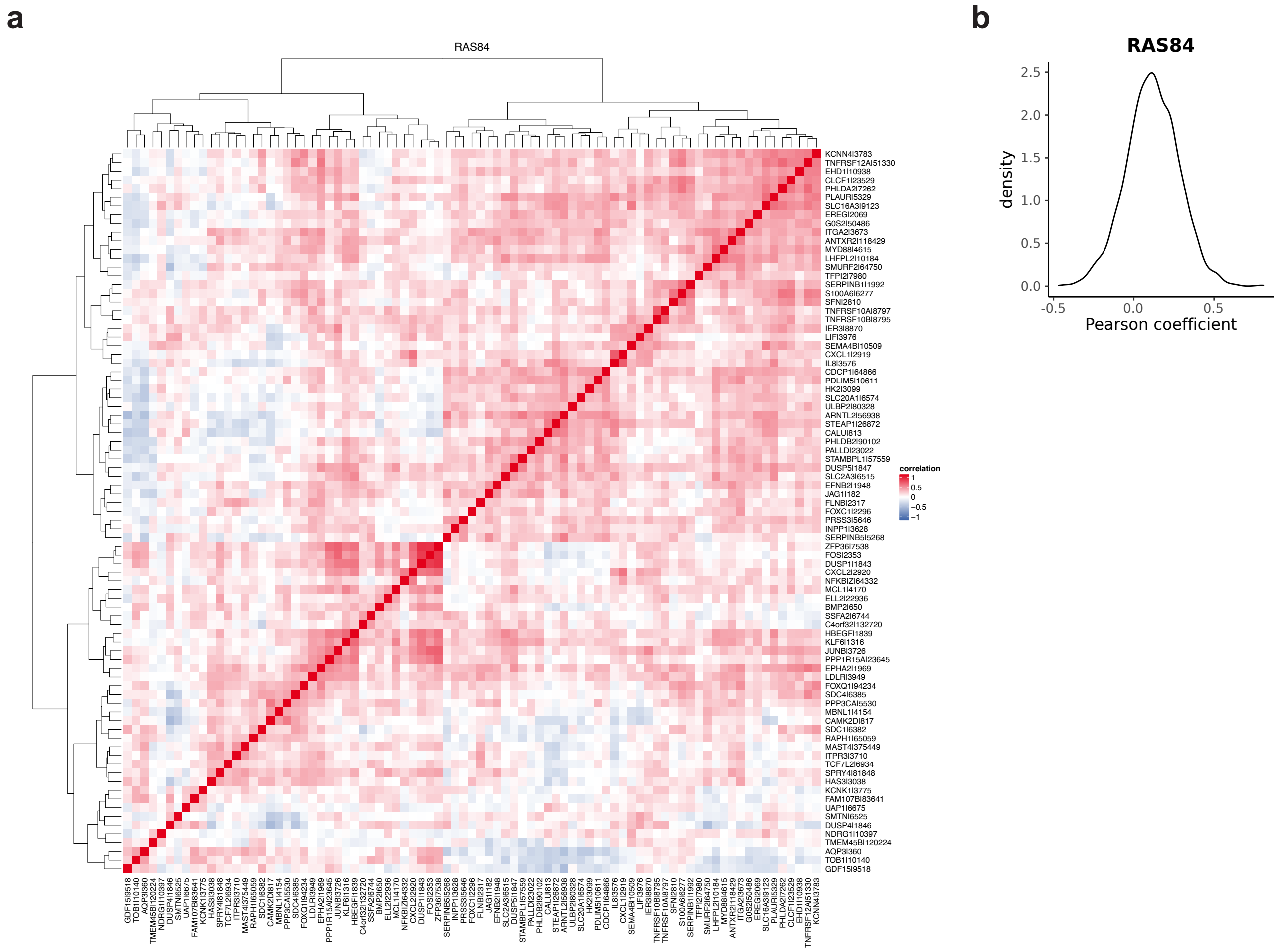

Supplementary Figure 10. (a) Heatmap showing the pairwise Pearson correlation coefficients for all RAS84 genes in the TCGA LUAD cohort. **(b)** The distribution of pairwise Pearson correlation coefficients in the TCGA LUAD cohort.

Supplementary figure 11

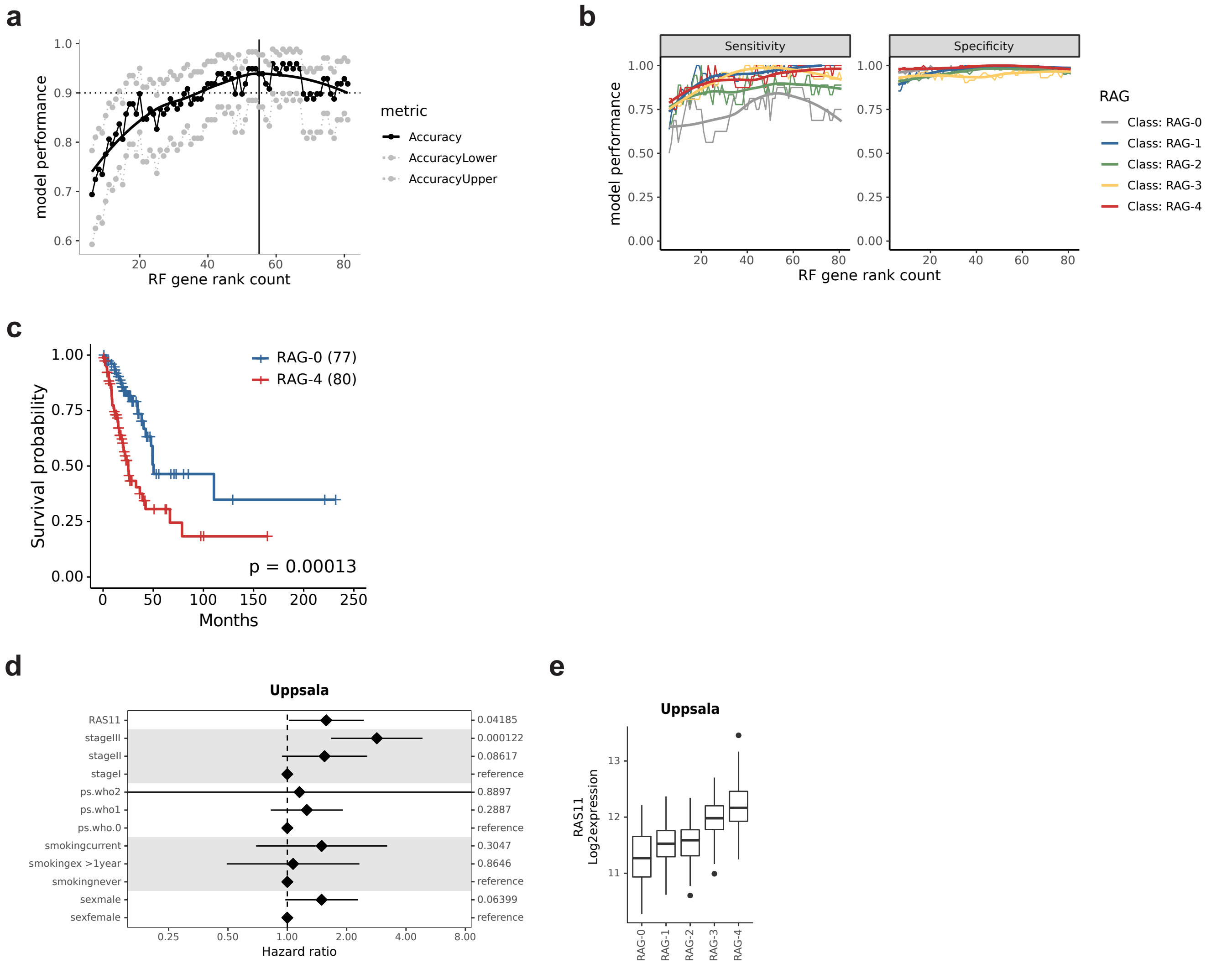

Supplementary Figure 11. (a) SVM classifier accuracy values plotted against the RAS84 rank number of genes used in the classifier construction. The gene rank was derived from the importance rank reported by a RandomForest model fit to all RAS84 genes using RAG labels and expression data from the TCGA LUAD cohort. The accuracy values were obtained by running the SVM classifier against a 20% test subset of the TCGA cohort. **(b)** SVM classifier sensitivity and specificity values plotted against the RAS84 rank number of genes used in the classifier construction as in (a). **(c)** Kaplan-Meier plot showing overall survival data from the TCGA LUAD cohort for patients from RAG-4 and RAG-0. The RAGs were derived from an SVM classification constructed using the top 55 performing RAS84 genes (a) (coxph p-value 0.00013), the number of patients per group is indicated in brackets. **(d)** Forest plots showing results from a multivariate Cox proportional-hazards analysis of OS in the Uppsala lung adenocarcinoma cohort (n=103 patients). The Coxph-MIC derived 11 gene signature mean values were tested for association with OS. TNM stage, World Health Organization (WHO) performance status, smoking history, gender and age were also tested. **(e)** Mean log2 expression distributions across Uppsala RAG groups calculated from the CoxphMIC derived 11 gene signature (RAG-0 n=15, RAG-1 n=33, RAG-2 n=45, RAG-3 n=64, RAG-4 n=37). The boxes show the median and IQR, the whiskers indicate +/- 1.5 x IQR, outliers lie outside this range.

Supplementary figure 12

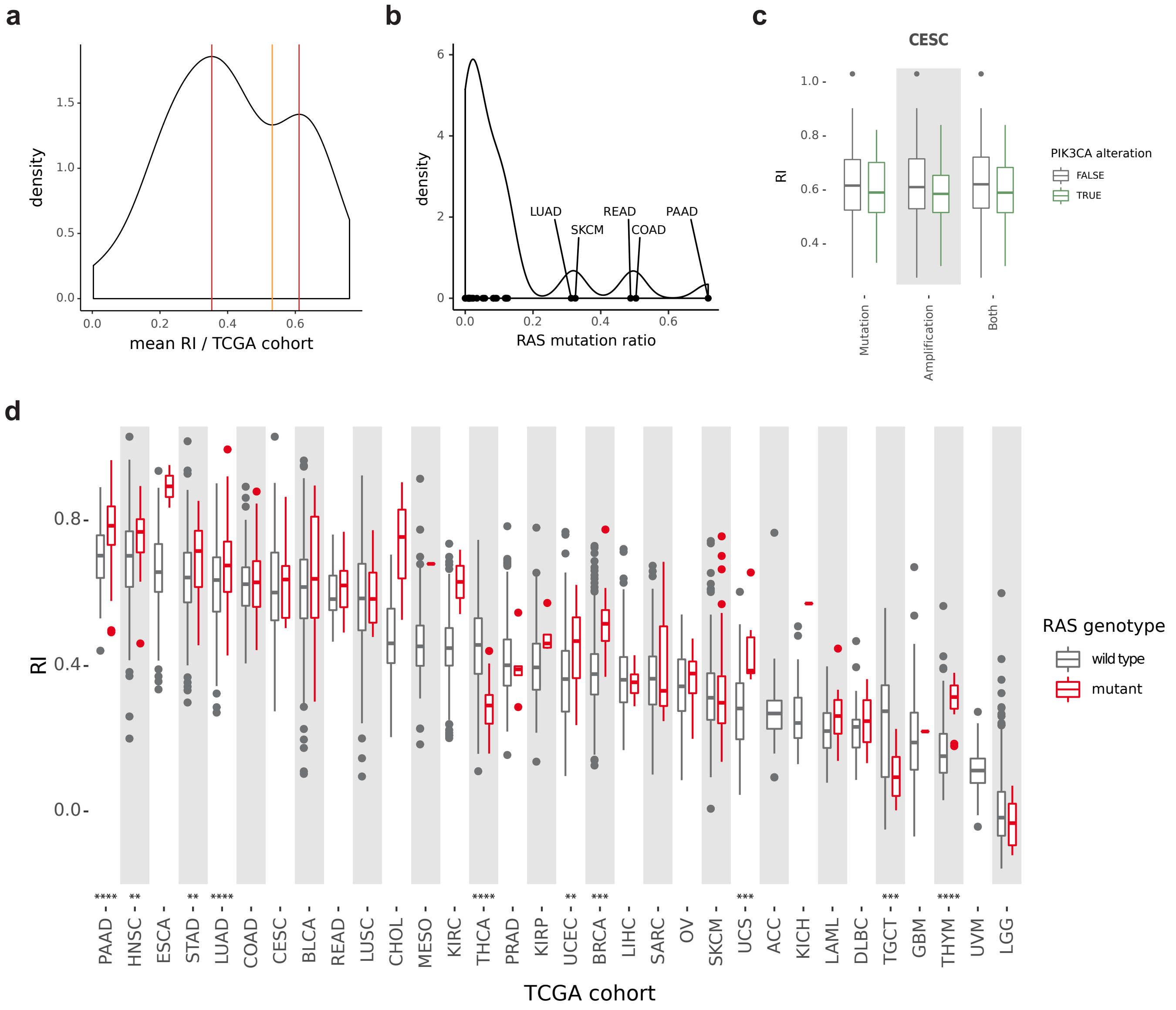

Supplementary Figure 12. (a) The distribution of mean RI values per cohort. The bimodal maxima are indicated by the red lines, the minima, segmenting the two populations of RAS activity cancers is indicated by the orange line. **(b)** The distribution of RAS (K, H and N) mutation frequencies in the different TCGA cohorts. **(c)** Boxplots showing RI distributions split by PIK3CA mutation, amplification and combined status for CESC (PIK3CA mutation(mut) n=77, mutation(wt) n=193, amplification(mut) n=40, amplification(wt) n=320, both(mut) n=104, both(wt) n=166). The boxes show the median and IQR, the whiskers indicate the largest/smallest) value </> the upper/lower) quartile +/- 1.5 * IQR, outliers lie outside this range. **(d)** Boxplots showing RI distributions per TCGA cohort split by RAS mutation status. Significant differences in RI means between the RAS mutant and wild-type groups are indicated by the stars at the bottom of the plot (Wilcoxon, ****<0.0001, ***<0.001, **<0.01). The boxes show the median and IQR, the whiskers indicate +/- 1.5 x IQR, outliers lie outside this range. (See Supplementary table 10 for n number of patients per group).
