# Supplementary material for: RAS oncogenic activity predicts response to chemotherapy and outcome in lung adenocarcinoma
Source: Nat Commun. 2022 Sep 26;13:5632. doi: 10.1038/s41467-022-33290-0 (PMC9512813; doi:10.1038/s41467-022-33290-0)
Supplement: Supplementary file 2 — Description to Additional Supplementary Information [file 41467_2022_33290_MOESM2_ESM.pdf]

Description of Additional Supplementary Files

1 - RAS signature details East et al.

2 - RAS84 genes

3 - GO_RAShigh_vs_RASlow

4 - RAS84_GDSC_drugs

5 - RAS84_CTRP_drugs

6 - GO_RAG_XvsRAG_0

7 - RAS84_heterogeneity

8 - coxph_RSG_results

9 - RAS84 ranked genes

10 - RASindex_RASmut_wilc

11 - Control signatures
