# Supplementary material for: RAS oncogenic activity predicts response to chemotherapy and outcome in lung adenocarcinoma
Source: Nat Commun. 2022 Sep 26;13:5632. doi: 10.1038/s41467-022-33290-0 (PMC9512813; doi:10.1038/s41467-022-33290-0)
Supplement: Supplementary file 4 — Reporting Summary [file 41467_2022_33290_MOESM4_ESM.pdf]

nature portfolio

Corresponding author(s): Julian Downward, Sophie de Carne Trecesson

Last updated by author(s): 30/08/22

Reporting Summary

Nature Portfolio wishes to improve the reproducibility of the work that we publish. This form provides structure for consistency and transparency in reporting. For further information on Nature Portfolio policies, see our Editorial Policies and the Editorial Policy Checklist.

Statistics

For all statistical analyses, confirm that the following items are present in the figure legend, table legend, main text, or Methods section.

n/a	Confirmed	
☐	☒	The exact sample size (*n*) for each experimental group/condition, given as a discrete number and unit of measurement
☒	☐	A statement on whether measurements were taken from distinct samples or whether the same sample was measured repeatedly
☐	☒	The statistical test(s) used AND whether they are one- or two-sided<br>*Only common tests should be described solely by name; describe more complex techniques in the Methods section.*
☐	☒	A description of all covariates tested
☐	☒	A description of any assumptions or corrections, such as tests of normality and adjustment for multiple comparisons
☐	☒	A full description of the statistical parameters including central tendency (e.g. means) or other basic estimates (e.g. regression coefficient) AND variation (e.g. standard deviation) or associated estimates of uncertainty (e.g. confidence intervals)
☐	☒	For null hypothesis testing, the test statistic (e.g. *F*, *t*, *r*) with confidence intervals, effect sizes, degrees of freedom and *P* value noted<br>*Give P values as exact values whenever suitable.*
☒	☐	For Bayesian analysis, information on the choice of priors and Markov chain Monte Carlo settings
☐	☒	For hierarchical and complex designs, identification of the appropriate level for tests and full reporting of outcomes
☐	☒	Estimates of effect sizes (e.g. Cohen's *d*, Pearson's *r*), indicating how they were calculated

Our web collection on statistics for biologists contains articles on many of the points above.

Software and code

Policy information about availability of computer code

Data collection	GEOquery_2.54.1
Data analysis	All code used in this manuscript is available at https://github.com/FrancisCrickInstitute/RAS84 limma_3.40.2<br>clusterProfiler _3.12.0<br>DESeq2_1.20.0)<br>Goseq_l.36.0<br>survival_3.l-ll.<br>survminer_0.4.4<br>coxphMIC_0.1.0<br>R_3.6.0

For manuscripts utilizing custom algorithms or software that are central to the research but not yet described in published literature, software must be made available to editors and reviewers. We strongly encourage code deposition in a community repository (e.g. GitHub). See the Nature Portfolio guidelines for submitting code & software for further information.

Data

Policy information about availability of data

All manuscripts must include a data availability statement. This statement should provide the following information, where applicable:

- Accession codes, unique identifiers, or web links for publicly available datasets
- A description of any restrictions on data availability
- For clinical datasets or third party data, please ensure that the statement adheres to our policy

The TRACERx tumour region gene-level RNA sequencing count data of 84 genes used during this study are available through the Cancer Research UK & University College London Cancer Trials Centre (ctc.tracerx@ucl.ac.uk) for non-commercial research purposes, and access will be granted upon review of a project proposal that will be evaluated by a TRACERx data access committee and entering into an appropriate data access agreement subject to any applicable ethical approvals. The TEMPUS data is commercially available from TEMPUS Lab (https://www.tempus.com/life-sciences/data-collaborations/#contact). Affymetrix data that was used to generate the KRASG13D134 signature have been deposited in GEO with the accession code GSE199871 [https://www.ncbi.nlm.nih.gov/geo/query/acc.cgi?acc=GSE199871]. The Uppsala cohort data used in this study are available in the GEO database under accession code GSE81089 [https://www.ncbi.nlm.nih.gov/geo/query/acc.cgi?acc=GSE81089]. The Seoul cohort data used in this study are available in the GEO database under accession code GSE40419 [https://www.ncbi.nlm.nih.gov/geo/query/acc.cgi?acc=GSE40419]. The Lambreth data used in this study are available in the ArrayExpress database under accession code E-MTAB-6149 [https://www.ebi.ac.uk/arrayexpress/experiments/E-MTAB-6149/]. All other data associated with this study are present in the paper or supplementary materials, or as cited.

Field-specific reporting

Please select the one below that is the best fit for your research. If you are not sure, read the appropriate sections before making your selection.

☒ Life sciences ☐ Behavioural & social sciences ☐ Ecological, evolutionary & environmental sciences

For a reference copy of the document with all sections, see nature.com/documents/nr-reporting-summary-flat.pdf

Life sciences study design

All studies must disclose on these points even when the disclosure is negative.

Sample size	Sample size was limited by the availability of patients meeting the criteria defined in our method.
Data exclusions	We did not exclude any data
Replication	We validated our observations in independent cohorts of patients when available. Only one of our results was not repeated because of the lack of required data in the literature. All other correlations were repeated in at least two cohorts, all of which were successful.
Randomization	When clinical data were available, we performed multivariate analyses to consider covariates
Blinding	Our exploratory meta-analysis used data that were not collected by us but by the groups we reference in the method and data availability section. The analysis was performed by one analyst who defined the groups and tested correlation with outcome, and response to therapy in the groups. A complete blind analysis was not possible. However, the data collection was blind to us because performed by other groups. After defining the patient groups, we performed outcome and response to therapy analyses and did not change our method to group the patients.

Reporting for specific materials, systems and methods

We require information from authors about some types of materials, experimental systems and methods used in many studies. Here, indicate whether each material, system or method listed is relevant to your study. If you are not sure if a list item applies to your research, read the appropriate section before selecting a response.

Materials & experimental systems

n/a	Involved in the study	
☒	☐	Antibodies
☒	☐	Eukaryotic cell lines
☒	☐	Palaeontology and archaeology
☒	☐	Animals and other organisms
☒	☐	Human research participants
☒	☐	Clinical data
☒	☐	Dual use research of concern

Methods

n/a	Involved in the study	
☒	☐	ChIP-seq
☒	☐	Flow cytometry
☒	☐	MRI-based neuroimaging
